# Spheroid trilineage differentiation model of primary mesenchymal stem/stromal cells under hypoxia and serum-free culture conditions

**DOI:** 10.3389/fbioe.2024.1444363

**Published:** 2024-07-31

**Authors:** Julia Moldaschl, Farhad Chariyev-Prinz, Stefan Toegel, Maike Keck, Ursula Hiden, Dominik Egger, Cornelia Kasper

**Affiliations:** ^1^ Institute of Cell and Tissue Culture Technologies, BOKU University, Vienna, Austria; ^2^ Karl Chiari Lab for Orthopaedic Biology, Department of Orthopedics and Trauma Surgery, Medical University of Vienna, Vienna, Austria; ^3^ Department of Plastic, Reconstructive and Aesthetic Surgery, Agaplesion Diakonieklinikum Hamburg, Hamburg, Germany; ^4^ Klinik für Plastische Chirurgie, Universität zu Lübeck, Lübeck, Germany; ^5^ Department of Obstetrics and Gynecology, Medical University of Graz, Graz, Austria; ^6^ Institute of Cell Biology and Biophysics, Leibniz University Hannover, Hannover, Germany

**Keywords:** mesenchymal stem cells, trilineage differentiation, spheroid, hypoxia, serum-free culture

## Abstract

Due to their unique properties, human mesenchymal stem/stromal cells (MSCs) possess tremendous potential in regenerative medicine, particularly in cell-based therapies where the multipotency and immunomodulatory characteristics of MSCs can be leveraged to address a variety of disease states. Although MSC-based cell therapeutics have emerged as one of the most promising medical treatments, the clinical translation is hampered by the variability of MSC-based cellular products caused by tissue source-specific differences and the lack of physiological cell culture approaches that closely mimic the human cellular microenvironment. In this study, a model for trilineage differentiation of primary adipose-, bone marrow-, and umbilical cord-derived MSCs into adipocytes, chondrocytes and osteoblasts was established and characterized. Differentiation was performed in spheroid culture, using hypoxic conditions and serum-free and antibiotics-free medium. This platform was characterized for spheroid diameter and trilineage differentiation capacity reflecting functionality of differentiated cells, as indicated by lineage-specific extracellular matrix (ECM) accumulation and expression of distinct secreted markers. The presented model shows spheroid growth during the course of differentiation and successfully supports trilineage differentiation for MSCs from almost all tissue sources except for osteogenesis of umbilical cord-derived MSCs. These findings indicate that this platform provides a suitable and favorable environment for trilineage differentiation of MSCs from various tissue sources. Therefore, it poses a promising model to generate highly relevant biological data urgently required for clinical translation and therefore might be used in the future to generate *in vitro* microtissues, building blocks for tissue engineering or as disease models.

## 1 Introduction

Due to their unique properties, including multilineage differentiation, immunomodulatory capacity (Diehl et al., 2017), and well-established isolation procedures (Martin et al., 2019), MSCs are of great interest for application in regenerative medicine and cell-based therapies. This has become a highly promising emerging field (Zhuang et al., 2021; Wright et al., 2021), with more than 1200 MSC-based clinical trials registered (https://clinicaltrials.gov/; accessed 21.05.2024) and more than 27 products containing MSCs approved worldwide (Teale et al., 2023). However, ensuring the clinically relevant quality of MSC-based cellular products and thus their safe and effective clinical translation remains a challenge. In fact, a major obstacle is the variability of MSC-based cellular products caused by tissue source-specific differences that lead to functional variations resulting in heterogeneous therapeutic efficacy (Mendicino et al., 2014; Yen et al., 2023). Another fundamental problem is the limited biological relevance of data obtained from conventional cell culture systems due to their inability to sufficiently recapitulate the human *in vivo* situation. This constraint ultimately raises the urgent need for optimized MSC cultivation approaches that closely mimic the human cellular microenvironment (Nikolits et al., 2021). Over the last decades, the awareness of the relevance of those advanced culture systems and the associated increase in biological reliability has grown tremendously. Aspects of optimized cell culture conditions include i) three-dimensional (3D) constructs instead of traditional two-dimensional (2D) cell culture formats (Saleh et al., 2012; Duval et al., 2017; Chaicharoenaudomrung et al., 2019), ii) reduced oxygen levels, considered as hypoxia (Egger et al., 2017; Zhao AG. et al., 2020; Yang et al., 2022) and iii) the absence of fetal bovine serum (FBS) (Palombella et al., 2022) and antibiotics (Llobet et al., 2015; Skubis et al., 2017; Li and Yue, 2019) as medium supplements. Positive effects of these advanced culture conditions over conventional culture systems have been reported in the literature. In fact, MSCs cultured in 3D systems as spheroids have been associated with enhanced matrix production (Duval et al., 2017), more potent paracrine effects, improved stemness, and better cell survival after transplantation (Yen et al., 2023). Furthermore, since MSC differentiation is largely controlled by their niche, 2D culture methods have critical limitations in regulating stem cell differentiation pathways resulting in low differentiation efficiency. MSCs cultured in spheroids, on the other hand, have been shown to have more robust adipo, osteo-, and chondrogenesis capabilities (Huang et al., 2011; Baraniak and McDevitt, 2012; Vidyasekar et al., 2016; Thakur et al., 2022).

Classically, FBS has been used as a culture supplement for MSCs. Nevertheless, there are apparent constraints associated with the utilization of FBS, particularly in the context of FBS-based cell culture products. In addition to recent regulatory restrictions in clinical settings and ethical concerns associated with the collection process, FBS poses significant scientific and safety challenges. In detail, the undefined and heterologous composition of animal origin components in FBS results in a high degree of heterogeneity and batch-to-batch variations of the product, which in turn causes changes in morphological, phenotypic, and population kinetic characteristics in MSCs produced. Moreover, in contrast to xenogenic serum-free alternatives, FBS poses a serious risk of pathogen transmission of zoonotic viral or prion diseases to MSCs and subsequently to their recipients, as well as xenoimmunization against bovine antigens (Spees et al., 2004; Gottipamula et al., 2013; Lee et al., 2022; Schepici et al., 2022). The internalization of xenogeneic antigens during FBS-supplemented culture is not eliminated even after post-harvest washing procedures of cell therapy products, which triggers a xenogeneic immune response. This affects the viability, safety, and efficacy of transplanted MSCs (Heiskanen et al., 2007; Lee et al., 2022). Consequently, the scientific interest in xenogenic serum-free culture medium supplements that are not associated with the aforementioned issues, has grown rapidly to implement the bench-to-bedside translation of MSC-based therapies. In comparison to the use of FBS, serum-free medium alternatives have shown to eliminate the risk of xenoimmunization and transmission of bovine pathogens. Among these, human platelet lysate (hPL) has recently been proposed as a physiologically relevant alternative. One advantage of hPL is its reduced batch-to-batch variability due to the nature of pooling the product from different blood donors (Palombella et al., 2022). In comparison to FBS, hPL has also been demonstrated to support proliferation (Gottipamula et al., 2013; Palombella et al., 2022) and to promote the immunomodulatory properties of MSCs (Mareschi et al., 2020).

Aminoglycoside antibiotics, commonly used in MSC cell culture, have been shown to affect cell proliferation (Skubis et al., 2017), and modulate the differentiation potential of MSCs, as they impede osteogenesis, chondrogenesis (Chang et al., 2006), and adipogenesis (Llobet et al., 2015; Goralczyk et al., 2017). The physiological relevance of hypoxic oxygen conditions for MSCs has been extensively investigated in the past, revealing several advantages regarding the therapeutic use of MSCs. When exposed to hypoxia, MSCs have been reported to exhibit increased proliferation, survival, migration (Yang et al., 2022), and enhanced chondrogenesis (Yasui et al., 2016; Ranmuthu et al., 2022). In addition, decreased oxygen levels have been shown to result in a more potent paracrine effect (Yang et al., 2022) and improved immunomodulation of MSCs (Kadle et al., 2018; Sarsenova et al., 2022).

Conventional cell culture systems for MSC spheroid formation have proven unable to provide the highly relevant biological data required for successful and safe clinical translation, as they do not sufficiently recapitulate the human *in vivo* situation when applying conventional culturing conditions.

Previous studies on MSC spheroid models have investigated the impact of hypoxic conditions (Munir et al., 2014; Yasui et al., 2016; Zubillaga et al., 2020) and serum-free culture employing hPL (Gardner et al., 2023) or chemically defined serum-free medium (Alimperti et al., 2014; Zhao Y. et al., 2020) on differentiation separately. However, to the best of our knowledge, there is no study on MSC spheroid multilineage differentiation models that focuses on the application of multiple of the aforementioned advanced culture conditions simultaneously.

The assessment of the trilineage differentiation potential of MSCs is of paramount importance for the comprehension of their capacity to develop into different cell types and for their utilization in regenerative medicine. The staining of differentiation-specific extracellular matrix components, including lipid vacuoles in preadipocytes and adipocytes for adipogenesis (Greenspan et al., 1985), calcium phosphate crystals as indicators for osteoblasts presence during osteogenesis (White et al., 2021), and matrix-bound sulfated glycosaminoglycans (sGAG) as chondrogenic markers (Templeton, 1988) are well-established techniques. A cost-effective and non-invasive approach or on-line monitoring of the differentiation process is the quantification of distinct differentiation markers secreted by the MSCs into the culture medium. To assess osteogenesis, a straightforward and rapid option is the activity measurement of Alkaline Phosphatase (ALP), a secreted enzyme associated with bone mineralization (Ansari et al., 2022). During adipogenic differentiation, levels of metabolic glycerol secretion can be determined. Glycerol, a product of lipolysis of triglycerides and glycerogenesis, is released into the cell culture medium by adipocytes (Rotondo et al., 2017; Graham et al., 2020). Secreted sGAG can be quantified as markers for chondrogenic differentiation employing the highly sensitive and widely used 1,9-dimethylmethylene blue (DMMB) assay (Templeton, 1988; Oke et al., 2003; Chariyev-Prinz et al., 2023).

In this study, a 3D MSC trilineage differentiation model under advanced culture conditions has been established to generate 3D microtissues that can potentially be used as disease models, *in vitro* tissues or building blocks for tissue engineering. A novel protocol was developed for the trilineage differentiation of adipose-, bone marrow- and umbilical cord-derived MSCs into adipocytes, chondrocytes and osteoblasts in media free of serum and antibiotics. MSCs were differentiated under hypoxia (5% O_2_) as spheroids in micropatterned multiwell plates. After several time points of differentiation, the spheroid diameter and the trilineage differentiation capacity reflecting the functionality of differentiated cells and expressed by lineage-specific ECM component accumulation and preservation of differentiation-specific trophic activities were analyzed and compared between the different MSC sources. To our knowledge, this is the first MSC spheroid trilineage differentiation model applying these four aspects of advanced cell culture conditions simultaneously.

## 2 Materials and methods

If not stated otherwise, reagents were purchased from Sigma-Aldrich, St. Louis, MO, United States.

### 2.1 Isolation and expansion of MSCs

#### 2.1.1 Adipose-derived MSCs

Human adipose tissue-derived MSCs (adMSCs) were isolated from a fat tissue resection 24 h after surgery from one donor, as described earlier (Egger et al., 2017). The use of human tissue was approved by the ethics committee of the University of Lübeck (reference number 20-333, November 2020). Briefly, the adipose tissue separated from the skin flaps was minced and digested with collagenase type IA at 37°C for 1 h, followed by a series of centrifugation and washing steps to obtain the stromal vascular fraction. This was then transferred to cell culture flasks (Sarstedt, Nümbrecht, Germany) and cultured in standard expansion medium consisting of MEM alpha (Thermo Fisher Scientific, Waltham, MA, United States), 0.5% gentamicin, 2.5% human platelet lysate (PL BioScience, Aachen, Germany) and 1 U/mL heparin (PL BioScience, Aachen, Germany) in a humidified incubator at 37°C, 5% CO_2_, and 5% O_2_. adMSCs were harvested when they reached approximately 80% confluence. Cells were detached using accutase and cryopreserved in cryomedium consisting of standard expansion medium as mentioned above, 10% hPL and 10% dimethyl sulfoxide in liquid nitrogen.

#### 2.1.2 Bone marrow-derived MSCs

Human bone marrow-derived MSCs (bmMSCs) were isolated from bone marrow obtained during hip arthroplasty from one donor. The use of human bone marrow was approved by the ethics committee of the Medical University of Vienna (reference number 2272/2020, January 2021). After surgery, bone marrow was transferred to cell culture flasks (TPP, Trasadingen, Switzerland) and cultured in expansion medium consisting of MEM alpha, 0.5% gentamicin, and 2.5% fibrinogen-depleted hPL (PL BioScience, Aachen, Germany) in a humidified incubator at 37°C, 5% CO_2_, and 21% O_2_. Cells were harvested at a confluence of approximately 80%. They were detached using TrypLE (Thermo Fisher Scientific, Waltham, MA, United States) and cryopreserved as described above.

#### 2.1.3 Umbilical cord-derived MSCs

Human umbilical cord-derived MSCs (ucMSCs) were isolated from umbilical cords acquired from accouchement from one donor; the tissue was stored at 4°C and processed within 24 h after the acquisition, as previously described (Majore et al., 2011). The use of human tissue was approved by the ethics committee of the Medical University of Graz (reference number 29-319 ex 16/17, July 2018).

For isolation, red blood vessels and cord blood were removed from the umbilical cords and the tissue was minced with scissors, transferred into cell culture flasks and cultured in standard expansion medium consisting of MEM alpha, 0.5% gentamicin, 2.5% human platelet lysate, and 1 U/mL heparin in a humidified incubator at 37°C, 5% CO_2_, and 5% O_2_. Cells were harvested and cryopreserved as described above.

### 2.2 Hypoxia reporter cell line

A hypoxia-responsive MSC reporter cell line (HRE-MSC) with a genetically encoded hypoxia sensor, generated and described by Schmitz et al. (2020), Schmitz et al. (2021), was used to determine the cellular response to an oxygen-reduced environment. In particular, hypoxia onset was visualized in spheroids, as shown in detail in Supplementary Figure S1. HRE-MSCs are based on the stabilization of hypoxia inducible factor 1α (HIF-1α) upon hypoxia, leading to the expression of the green fluorescent protein UnaG. The cells were thawed and expanded in MEM alpha, 0.5% gentamicin, and 10% human serum at 37°C, 5% CO_2_, and 21% O_2_. Subsequently, they were seeded into Sphericalplate 5D^®^ low attachment micropatterned 24-well plates (Kugelmeiers, Erlenbach, Switzerland) at a cell density of 5,00,000 cells per well, resulting in approximately 670 cells per spheroid. Spheroids formed after 24 h and medium was changed every 2–3 days. Starting from 21% O_2_, the oxygen level was gradually decreased until fluorescence was detected, indicating the onset of hypoxia.

### 2.3 Immunophenotyping

In order to comply with the cell surface marker panel for the minimal identification of human multipotent MSCs proposed by the International Society for Cellular Therapy (Dominici et al., 2006), we determined MSC-specific surface antigen expression of the cells used. adMSCs, bmMSCs, and ucMSCs at passage four were detached by accutase treatment and stained with a BD Stemflow™ Human MSC Analysis Kit (BD Biosciences, Franklin Lakes, NJ, United States) according to the manufacturer’s instructions. This kit detects the surface antigen markers CD73, CD90, and CD105, which must be expressed (≥95% positive), and the hematopoietic lineage markers CD34, CD45, CD11b, CD19, and HLA-DR, which must be absent (≤2% positive). Stained cells were resuspended in an appropriate volume of flow cytometry buffer [1% bovine serum albumin, 2 mM EDTA disodium salt dihydrate in phosphate-buffered saline (PBS, Gibco, Thermo Fisher Scientific, Waltham, MA, United States)]. Samples were examined on a CytoFLEX S4 followed by analysis using Kaluza Flow Cytometry software version 2.1 (both Beckman Coulter, Brea, CA, United States).

### 2.4 3D cell culture and trilineage differentiation

After thawing, adMSCs, bmMSCs, and ucMSCs were expanded in a humidified incubator at 37°C, 5% CO_2_, and 5% O_2_ in cell culture flasks (Sarstedt, Nümbrecht, Germany) in an antibiotic-free medium consisting of MEM alpha, 2.5% human platelet lysate and 1 U/mL heparin for up to four passages. Cells were then harvested by accutase treatment, counted and seeded into Sphericalplate 5D^®^ low attachment micropatterned 24-well plates at a cell density of 500,000 cells per well, resulting in approximately 670 cells per spheroid. After 24 h and the formation of spheroids, the antibiotic free medium mentioned above was replaced by the respective differentiation medium. Adipogenic differentiation was performed with MSCgo™ Adipogenic Differentiation Medium supplemented with MSCgo™ Adipogenic SF, XF Supplement Mix I and MSCgo™ Adipogenic SF, XF Supplement Mix II, osteogenic differentiation with MSCgo™ Osteogenic Differentiation Medium and chondrogenic differentiation with MSCgo™ Chondrogenic Differentiation Medium supplemented with MSCgo™ Chondrogenic Differentiation Supplement Mix. All supplements were mixed 1:10 with the respective basal medium. All differentiation media and respective supplements are xeno- and serum-free and were purchased from Sartorius, Göttingen, Germany. Differentiation was performed in a humidified incubator at 37°C, 5% CO_2_, and 5% O_2_ for a maximum of 21 days in the absence of antibiotics. The medium was changed every 2–3 days. The chosen hypoxic oxygen level is based on preliminary hypoxia onset analyses as shown in Supplementary Figure S1.

For all subsequent analyses, spheroids originating from three different wells were sampled for each MSC type and time point.

### 2.5 Spheroid diameter analysis

Light microscopic images of adipogenic, osteogenic and chondrogenic differentiation were taken using a Leica DMi1 at 200x magnification. At least 10 images per sample type were processed in FIJI (ImageJ software). To determine spheroid diameter, three different angles per spheroid were measured and averaged.

### 2.6 Differentiation-specific stains

Spheroids were harvested after 0, 7, 14 or 21 days of differentiation and washed with PBS. For adipogenic differentiation, spheroids were fixed with 4% paraformaldehyde for 1 h at 4°C and permeabilized with 0.1% Triton-X for 30 min at room temperature with constant agitation. The samples were then stained with Nile Red and DAPI at a final concentration of 50 µg/mL and 5 µg/mL respectively to visualize lipid droplets and cell nuclei, followed by a wash step with PBS. Osteogenic differentiated spheroids were fixed with 70% ethanol for 30 min at 4°C followed by incubation in Calcein staining solution (5 µg/mL) overnight at room temperature with agitation to assess calcium deposition in the extracellular matrix. Cell nuclei were then counterstained with DAPI at a concentration of 5 µg/mL for 40 min and samples were washed 6 times with PBS. Fluorescence staining of adipogenic and osteogenic differentiated spheroids was imaged using a Leica TCS SP8-STED laser scanning confocal microscope.

Chondrogenic spheroids were fixed in 4% paraformaldehyde for 1 h and incubated overnight in 30% sucrose in PBS with agitation. Samples were embedded in Tissue-Tek^®^ O.C.T. Compound (Sakura Finetek United States, Inc., Torrance, CA, United States) and snap frozen. Sections of 20 µm were cut using a Leica CM1860 cryostat. Frozen sections were then thawed at room temperature and post-fixed in 4% paraformaldehyde for 5 min. Slides were rinsed twice for 5 min with PBS and incubated with 3% acetic acid for 5 min. The sections were then stained with 1% Alcian blue in 3% acetic acid (pH 2.5) for 30 min to visualize sGAGs in the ECM. After rinsing with tap water three times for 3 min, the slides were counterstained with 0.1% Nuclear Fast Red solution for 5 min, rinsed three times for 2 min with ddH_2_O and dehydrated through a graded alcohol series. The sections were then processed for microscopy with Eukitt^®^ mounting medium and a coverslip and imaged using a Leica DMi1. For all three differentiation lines, samples collected on day 0 served as negative controls.

### 2.7 Semi-quantitative image analysis

Light and confocal microscopy images of samples processed as described in Section 2.6 were analyzed using FIJI (ImageJ) for the abundance and intensity of characteristic ECM molecules and nuclei signals. The ratio of characteristic ECM to nuclei signal and the area fraction of nuclei signal to total spheroid area were determined during the course of differentiation. An increasing value of the latter may indicate cell proliferation within the spheroid, while a decreasing nuclei-to-total spheroid area fraction may suggest ECM accumulation in complementation with other applied analyses. Three images per sample were processed and the values obtained were averaged. For each dye and nucleus counterstain, a threshold was determined and applied to the corresponding differentiation lineage. For adipogenic and osteogenic differentiation the red, blue and green signals were analyzed, while for chondrogenic differentiation, the colors were separated using the *Color Deconvolution 2* plug-in and their signals were measured. The exact methods are described in detail in Supplementary Figures S2, S3.

### 2.8 Secreted marker expression quantification

Cell culture medium was collected on days 0, 3, 7, 10, 14, 17, and 21 of trilineage differentiation and stored at −20°C until further analysis. For all three secreted marker analyses, medium collected on day 0 served as a blank control and was subtracted from all readings. Per condition, three wells containing 2000 µL of cell culture medium, respectively, were analyzed.

#### 2.8.1 Glycerol quantification

To quantify the amount of glycerol produced during adipogenic differentiation, 50 µL of cell culture medium per sample was analyzed for secreted glycerol. For the measurement, a YSI 2900D Biochemical Analyzer and a compatible Glycerol Kit were used as per manufacturer’s instructions (Kreienbaum, Langenfeld, Germany).

#### 2.8.2 ALP activity quantification

Cell culture medium collected during osteogenic differentiation was centrifuged for 5 min at 14,000 × *g* (4°C) and 80 µL were transferred to the wells of a 96-well plate. 20 μL of pNPP stock solution (one Tris buffer tablet and one SIGMAFAST™ p-nitrophenyl phosphate tablet dissolved in 4 mL ddH_2_O) was added to each well and the plate was incubated for 40 min at 37°C. Immediately, absorbance (405 nm) was measured using an Infinite M1000 plate reader (Tecan, Männedorf, Switzerland). The amount of p-nitrophenolate (product of the enzymatic reaction) was determined using a p-nitrophenolate calibration line. This standard consisted of 4-nitrophenol dissolved in Tris buffer (one Tris buffer tablet in 20 mL ddH_2_O) and was measured without prior incubation. The ALP activity U (µmol/min), which corresponds to the amount of enzyme that converts 1 µmol of substrate per minute, was then calculated using the following formula:

$$\text{ALP}-\text{activity }U=\left(\frac{c_{pNP}}{0.1391}*t^{-1}\right)$$

$c_{pNP}$ is the concentration of p-nitrophenolate (µg/mL), 0.1391 the conversion factor from µg/mL to µmol/L and t the incubation time (minutes). Consequently, U * V with V as the volume (L) gives the volumetric enzyme activity.

#### 2.8.3 sGAG quantification

In 96-well plates, 200 μL of DMMB solution [46 μM DMMB, 40 mM glycine, and 40 mM NaCl in ddH_2_O (pH 3.0)] was added to 20 μL of the collected medium during chondrogenic differentiation. The absorbance was measured at 530 nm and 590 nm on an Infinite M1000 plate reader and the ratio was calculated to determine the amount of sGAG, using chondroitin sulfate C as a standard.

### 2.9 Statistical analysis

All results are presented as mean ± standard deviation of at least three independent technical replicates of a single experiment. The sample size “n” of the experiment is given in the legend of each corresponding figure. Prior to statistical tests, the data was analyzed for normality using the Shapiro-Wilk test. To compare day 0/day 3 against day 21 values withing the same tissue origin we applied a paired t-test. Two-way analysis of variance (ANOVA) test followed by Tukey’s multiple comparisons test was performed to compare day 21 values among different tissue origins. Data were plotted and analyzed using GraphPad Prism 8.0.0 software for Windows (GraphPad Software, San Diego, CA, United States). Significance is indicated as follows: *p < 0.05, **p < 0.01, and ***p < 0.001.

## 3 Results

### 3.1 MSC specific surface marker expression

**FIGURE 1:**
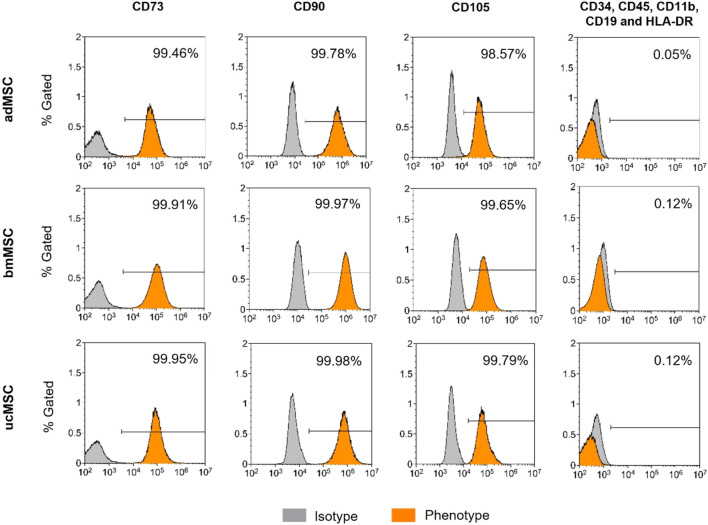
Immunophenotyping of adMCSs, bmMSCs, and ucMSCs. Cells examined were positive for CD73, CD90, and CD105 and negative for CD34, CD45, CD11b, CD19, and HLA-DR cell surface antigen expression, shown as % Gated, the *x*-axis shows signal intensity. Cells were detached at passage four and subsequently stained using the BD Stemflow™ Human MSC Analysis Kit. 100,000 events were recorded for adMSCs and bmMSCs and 40,000 events for ucMSCs. Samples were measured by flow cytometry (CytoFLEX S4) and analyzed using Kaluza Flow Cytometry software version 2.1.

In 2006, the International Society for Cellular Therapy proposed a cell surface marker panel as the minimal set for identification of human bone marrow-derived MSCs (Dominici et al., 2006). MSCs must express CD73, CD90, and CD105 (≥95% positive), and lack expression of CD34, CD45, CD14 or CD11b, CD79α or CD19 and HLA-DR antigen markers (≤2% positive). To comply with this minimal set of cell surface marker panel, the expression level of positive and negative antigen markers was determined in the cells used. Flow cytometric analysis confirmed the expression of specific positive (≥98.57%) and negative surface markers (≤0.12%) in the required range (Figure 1). No significant differences in the expression patterns of the markers tested were found between the tissue sources. These results suggest that the tested MSCs correspond to the minimal set of surface markers for the definition of human MSCs. Hence, this analysis suggests that the tested MSCs are suitable for serving as a robust foundation for this model, demonstrating their capability to exhibit characteristic MSC properties.

#### 3.1.1 Spheroid diameter and nuclei area fraction

**FIGURE 2:**
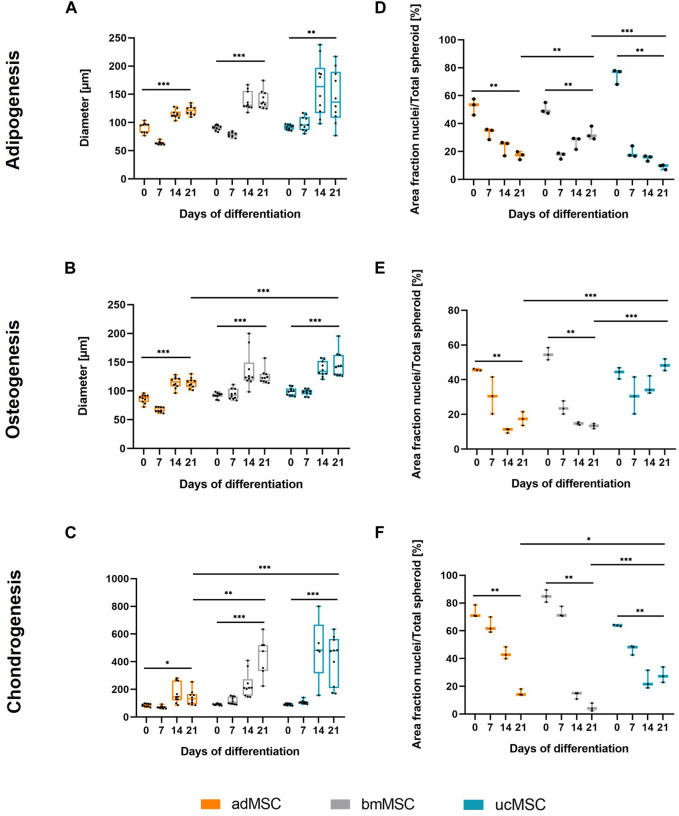
Spheroid diameter (**A–C**) and area fraction of nuclei (**D–F**) after 0, 7, 14, and 21 days of trilineage differentiation of MSCs from different tissue sources. **(A + D)**, Adipogenic differentiation; **(B + E)**, Osteogenic differentiation; **(C + F)**, Chondrogenic differentiation. Microscope images of the spheroids were captured and analyzed in ImageJ. In order to evaluate the area fraction of nuclei signal to total spheroid area, the images were semi-quantitatively analyzed as described in Supplementary Figure S2. All values represent the mean of 10 independently measured spheroids (spheroid diameter), or three independently measured spheroids (nuclei area fraction) generated in a single experiment. The whiskers indicate the maximum and minimum values. Prior to statistical tests, the data was analyzed for normality using the Shapiro-Wilk test. Statistical analysis was performed by paired t-test (day 0 against day 21 values) and two-way analysis of variance (ANOVA) for comparison of day 21 values among the tissue sources. For the latter, significance was determined using Tukey’s multiple comparisons test (*p ≤ 0.05; **p ≤ 0.01; ***p ≤ 0.001).

To characterize the proposed 3D trilineage differentiation model, we examined the spheroid size, the nuclei area fraction, and the trilineage differentiation capacity of MSCs from various sources. This comprised analyses of deposited components in the ECM and secreted markers specific for each differentiation lineage after defined timepoints of differentiation. The spheroid diameter and the area fraction of the nuclei of adMSCs, bmMSCs and ucMSCs were measured after 0, 7, 14, and 21 days of trilineage differentiation by analyzing light and confocal microscopy images of respective spheroids (Figure 2). In general, this spheroid size analysis (Figures 2A–C) revealed crucial differences among the tissue sources. Except for the chondrogenic lineage, ucMSCs generated the highest spheroid diameter after 21 days of differentiation (adipogenesis: 146 µm ± 44 μm, osteogenesis: 146 µm ± 21 µm), followed by bmMSCs (adipogenesis: 140 µm ± 15 μm, osteogenesis: 125 µm ± 13 µm), while adMSC spheroids showed the smallest diameter (adipogenesis: 122 µm ± 8 μm, osteogenesis: 115 µm ± 8 µm). Interestingly, a significant increase in spheroid diameter from day 0 to day 21 could be observed for all tested MSC types and differentiation lineages. In fact, during adipogenesis (Figure 2A) the spheroid diameter of adMSCs grew from 91 µm ± 8 μm to 122 µm ± 8 μm, bmMSCs grew from 91 µm ± 4 μm to 140 µm ± 15 µm, and ucMSCs from 92 µm ± 4 μm to 146 µm ± 44 µm. Figure 2B shows spheroid diameter differences for osteogenic differentiation. In detail, adMSCs exhibited an increase of 86 µm ± 7 μm to 114 µm ± 8 μm, while bmMSCs increased from 92 µm ± 5 μm to 125 µm ± 12 μm, and the spheroid diameter of ucMSCs increased from 100 µm ± 6 μm to 146 µm ± 21 µm. Figure 2C shows the increase of spheroid diameter during chondrogenic differentiation of adMSCs (86 µm ± 10 μm to 155 µm ± 67 µm), bmMSCs (92 µm ± 5 μm to 358 µm ± 106 µm), and ucMSCs (91 µm ± 7 μm to 418 µm ± 162 µm).

The nuclei area fraction (Figures 2D–F) declined between days 0 and 21 of differentiation across all three tissue sources, with the exception of ucMSCs during osteogenesis. In this condition (Figure 2E), the nuclei share declines after d0 (43.9% ± 2.7%) and then increases again upon day 7 (30.8% ± 8.7%) until day 21 (48.5% ± 2.8%), suggesting proliferation upon day 7. During the adipogenic differentiation, bmMSCs exhibited a comparable pattern (Figure 2D), although the nuclei area of day 21 (32.9% ± 3.9%) did not exceed the level of day 0 (50.5% ± 3.3%) and even displayed a significant decrease. While during adipogenesis bmMSCs showed the smallest average difference among the tissue sources between day 0 and day 21 (17.7% compared to 30.5% in adMSCs and 65.4% in ucMSCs), they displayed the greatest difference and steepest decrease in nuclei share in this timeframe in the chondrogenic differentiation (Figure 2F). In detail, bmMSCs showed an average difference of 80.3%, while adMSCs differed by 58.1%, and ucMSCs differed by 35.8%. The reduction in nuclei area fraction measured in the course of differentiation and considered as a relative value accompanied by an increase of the spheroid size can be indicative of ECM accumulation and, consequently, successful differentiation. The integrated assessment of spheroid size, nuclei share, ECM accumulation, and differentiation marker secretion provides robust insights into the differentiation status of MSCs during the differentiation process.

### 3.2 Differentiation-specific ECM component accumulation

**FIGURE 3:**
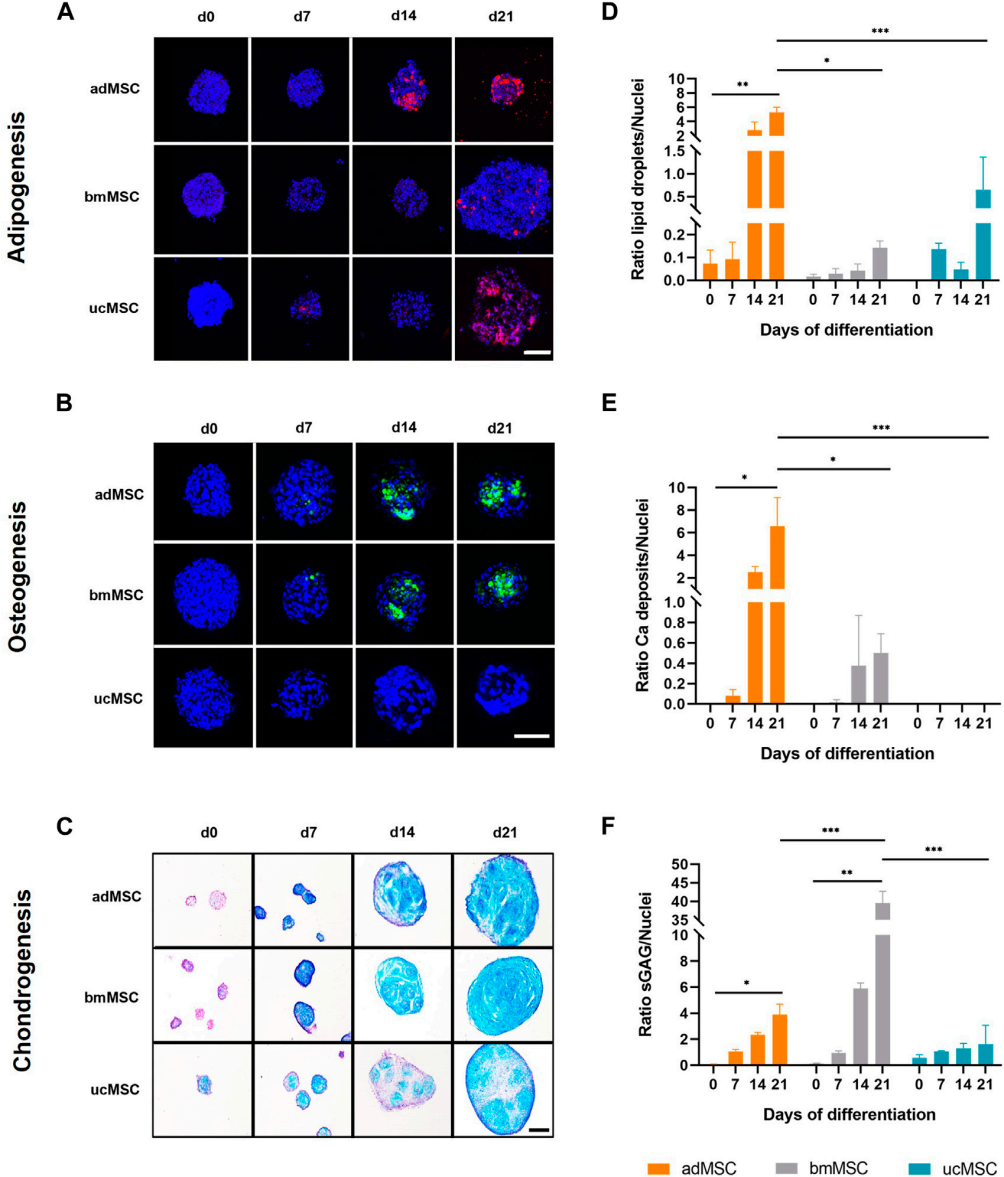
Analyses of lineage-specific ECM component accumulation. Spheroids formed of MSCs from three different tissue sources were analyzed after 0, 7, 14, and 21 days of trilineage differentiation. Qualitative analyses by (fluorescence) staining are shown in **(A–C)**. For adipogenic differentiation **(A)** lipid droplets were stained with Nile Red and visualized in red. During osteogenesis **(B)** calcium phosphate deposits were monitored and labelled with Calcein in green. For both adipogenic and osteogenic differentiation cell nuclei were counterstained with DAPI, shown in dark blue. Fluorescence staining of adipogenic and osteogenic differentiated spheroids was imaged by scanning confocal microscopy. Scale bar 100 µm each. Chondrogenically differentiated spheroids **(C)** were cryosectioned and sGAG were stained with Alcian Blue, while cell nuclei were stained in purple with Nuclear Fast Red. Images were taken by light microscopy. Scale bar 200 µm. The images shown are one representative out of three images taken, respectively. Semi-quantitative analysis of the specific ECM staining is shown in **(D–F)** for adipogenic, osteogenic and chondrogenic differentiation, respectively. Detailed analysis procedure is described in Supplementary Figure S3. A maximum projection of all z-stacks was generated. All image stacks were previously acquired with a 20× magnification objective at a resolution of 1,024 × 1,024 pixels, scan speed 5 μs, z-step size of 2 μm across 60 μm. For all three differentiation lineages, samples harvested on day 0 served as negative control. All values represent the mean of three independently imaged and subsequently analyzed spheroids generated in a single experiment, the error bars indicate the standard deviation. Prior to statistical tests, the data was analyzed for normality using the Shapiro-Wilk test. Statistical analysis was performed by paired t-test (day 0 against day 21 values) and two-way analysis of variance (ANOVA) for comparison of day 21 values among the tissue sources. For the latter, significance was determined using Tukey’s multiple comparisons test (*p ≤ 0.05; **p ≤ 0.01; ***p ≤ 0.001).

Next, the characteristic ECM component accumulation for each differentiation lineage was determined in the spheroids after 0, 7, 14, and 21 days of trilineage differentiation (Figure 3). For adipogenesis, lipid vacuoles were visualized with Nile Red (Figure 3A), while for osteogenic differentiation calcium phosphate crystal deposition was verified with Calcein staining (Figure 3B). Chondrogenic differentiation was monitored by staining sGAG with Alcian Blue (Figure 3C). This analysis showed that our model supported differentiation-specific ECM component accumulation after 21 days of MSC trilineage differentiation from all three tissue sources, with the exception of osteogenic differentiation of ucMSCs. In this particular case, no calcium phosphate was deposited even after 21 days of differentiation (Figures 3B, E). The data revealed significant differences in the level of characteristic ECM component expression between tissue sources and differentiation lineages in this model. For adipogenesis (Figures 3A, D), adMSCs showed the highest increase in lipid droplet production during differentiation, with a significantly higher ECM to cell nucleus ratio at day 21 (5.3 ± 0.6) compared to ucMSCs (0.9 ± 0.8) and bmMSCs (0.1 ± 0.02), which showed the lowest levels of lipid droplet accumulation. The low levels of specific ECM accumulation in the bmMSCs are reflected in the nuclei area fraction pattern (Figure 2D). Besides the observation that ucMSCs showed negligible calcium phosphate deposition during osteogenesis, higher calcium phosphate deposition for adMSCs (6.6 ± 2.1) compared to bmMSCs (0.5 ± 0.2) could be observed (Figures 3B, E). During chondrogenesis (Figures 3C, F), spheroids formed by bmMSCs expressed the highest sGAG to background ratio after 21 days of differentiation (39.6 ± 2.6), ucMSCs showed the lowest sGAG background ratio (1.6 ± 1.2) compared to the other tissue sources.

### 3.3 Quantification of secreted markers

**FIGURE 4:**
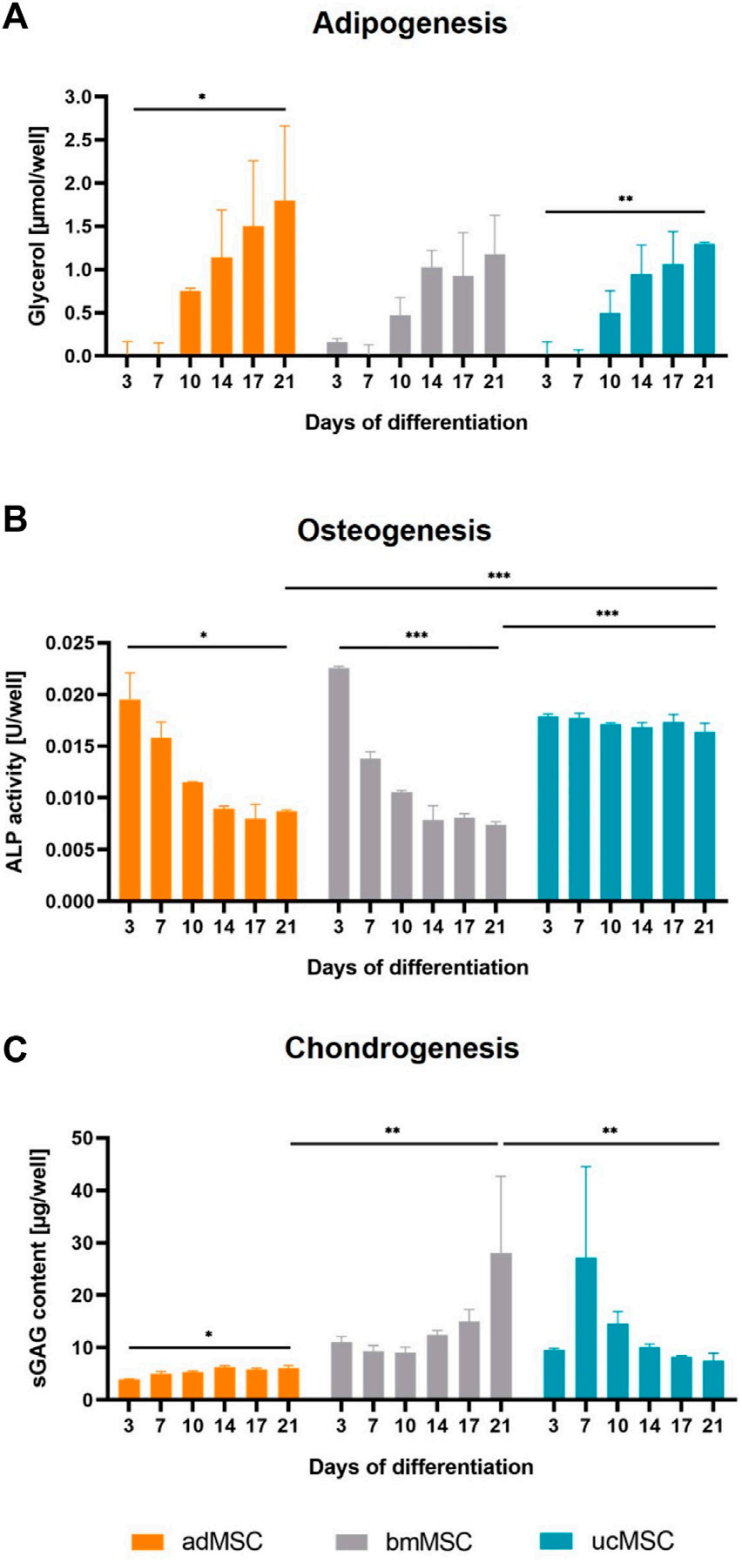
Quantification of secreted differentiation markers. Cell culture medium was analyzed after 3, 7, 10, 14, 17, and 21 days of trilineage differentiation of MSCs from different tissue sources. Glycerol expression was quantified for adipogenesis **(A)**, ALP activity for osteogenesis **(B)** and secreted sGAG expression for chondrogenesis **(C)**. Medium collected on day 0 served as blank control and was subtracted from all measurements. All values are the mean of three independently measured wells of a single experiment. Error bars indicate standard deviation. Prior to statistical tests, the data was analyzed for normality using the Shapiro-Wilk test. Statistical analysis was performed by paired t-test (day 0 against day 21 values) and two-way analysis of variance (ANOVA) for comparison of day 21 values among the tissue sources. For the latter, significance was determined using Tukey’s multiple comparisons test (*p ≤ 0.05; **p ≤ 0.01; ***p ≤ 0.001).

We quantified lineage-specific marker secretion in the cell culture medium after 3, 7, 10, 14, 17, and 21 days of trilineage differentiation of adMSCs, bmMSCs and ucMSCs. During adipogenesis, levels of metabolic glycerol secretion were determined, as shown in Figure 4A. The results revealed an increase of glycerol during the differentiation for all tissue sources, although this increase between day 3 and day 21 was only significant for adMSCs (0.01 ± 0.13 to 1.8 ± 0.7 µmol/well) and ucMSCs (0.02 ± 0.12 to 1.30 ± 0.02 µmol/well). Furthermore, adMSCs showed the highest levels of secreted glycerol after 21 days of differentiation. These findings complement the observation in Figure 3, thus supporting robust adipogenesis of adMSCs compared to other sources. For osteogenesis, ALP activity was quantified in the cell culture medium, as shown in Figure 4B. At day 3 of differentiation, the ALP activity of both adMSCs and bmMSCs showed a peak of 0.020 and 0.022 U/well, respectively, and decreased during further osteogenic differentiation. For ucMSCs, no significant change in ALP activity was detected between day 3 and day 21 of osteogenic differentiation, reflecting the above observations of negligible calcium phosphate deposition by ucMSCs (Figures 3B, E) and the increasing nuclei share (Figure 2E) during osteogenesis. Taken together, these findings suggest limited osteogenic differentiation of the ucMSCs used in this specific model. Figure 4C shows the levels of secreted sGAG during chondrogenic differentiation. Consistent with the results of the nuclei area fraction assessment (Figure 2F) and the sGAG accumulation analyses (Figures 3C, F), bmMSCs showed an increase in secreted sGAG levels between day 3 and day 21 of differentiation (11.0 ± 0.9 to 28.1 ± 11.9 µg/well) and a higher secreted sGAG expression on day 21 compared to adMSCs (6.1 ± 0.4 µg/well) and ucMSCs (7.5 ± 1.2 µg/well), suggesting a superior chondrogenic capacity of bmMSCs among the tissue sources. ucMSCs expressed a peak of secreted sGAG of 27.2 ± 14.2 µg/well on day 7, with a further decrease until day 21. This result is consistent with the findings of ECM accumulation, where no considerable sGAG levels were detected between day 0 and 21 (Figure 3C).

## 4 Discussion

In this study, a scaffold-free 3D trilineage differentiation model of MSCs derived from three different human tissues is described. To the best of our knowledge, this is the first comprehensive MSC differentiation platform cultured as spheroids under the application of multiple advanced culturing conditions, comprising a combination of hypoxic conditions and serum- and antibiotics-free medium composition. Existing studies on three-dimensional scaffold-free MSC differentiation systems have focused on either one (Wang et al., 2014; Vidyasekar et al., 2016; Thakur et al., 2022) or a few (Winter et al., 2003; Tsvetkova et al., 2021) tissue sources, primarily under the influence of only a single (Alimperti et al., 2014; Munir et al., 2014; Zhao Y. et al., 2020; Gardner et al., 2023) of the aforementioned advanced cell culture conditions.

The presented method of spheroid generation in microwell plates and trilineage differentiation using commercially available media is comprehensive, straightforward, user-friendly, and does not require the use of specialist equipment. Furthermore, the spheroid size can be scaled by varying the number of seeded cells per well. However, the increase of the spheroid size is intrinsically limited by necrosis (spheroid diameter > 500 µm) due to diffusion limitations for oxygen and nutrition (Bartosh et al., 2010; Ivanov et al., 2014). The design of the Sphericalplate 5D^®^ micropatterned multiwell plates enables the generation of 9,000 spheroids per 24-well plate, providing an excellent future potential for up-scaling. Next to the aforementioned features, the simplified procedures of spheroid generation, differentiation, media change and harvesting are posing major benefits of the herein presented differentiation platform compared to commonly employed spheroid differentiation platforms (Sphericalplate 5D, 2024). In detail, culture systems like the hanging drop method and round-bottom well plates, while effective, face challenges in scalability, consistency and labor intensity. Low-attachment plates offer good alternatives with high relevance and potential for up-scaling, though they do not provide precise control over the spheroid size, unless the substrates are patterned into small regions to produce one spheroid per region (Liu et al., 2021; Shen et al., 2021). Therefore, we consider the employed microwell platform a superior, balanced and highly relevant spheroid differentiation system. However, a potential limitation associated with buoyancy and fusion of spheroids in an undifferentiated state, should be considered when employing the platform. Due to this effect, morphological comparability with differentiated single spheroids was not given, leading to the decision to use day 0 spheroids as controls.

MSCs used for this study fulfilled the minimal criteria for defining human MSCs proposed by International Society for Cellular Therapy (Dominici et al., 2006), comprising plastic adherence and characteristic surface marker expression (Figure 1). Furthermore, successful trilineage differentiation and therefore functionality of the differentiated cells was indicated by increasing spheroid growth, a decreasing nuclei area fraction, lineage-specific ECM component accumulation and expression of selected differentiation markers could also be confirmed (Figures 2, 4), making employed MSCs suitable for the development of the proposed model.

Notably, MSCs derived from umbilical cord pose an exception as this cell population exhibited successful adipogenic and chondrogenic, but no osteogenic differentiation in our model. In contrast to the adipogenic and chondrogenic differentiation potential, which has been confirmed by several investigators, the osteogenic capacity of ucMSCs is controversially discussed. While several scientific groups describe the osteogenic potential comparable to bmMSCs (Diao et al., 2009; Hou et al., 2009), others demonstrate limited capacity of ucMSCs to undergo osteogenesis (Huang et al., 2009). Strikingly, Majore et al. reported poor osteogenic differentiation efficiency of enzyme-free isolated MSCs from whole umbilical cord tissue, even after the addition of a potent osteoinductive substance such as 1.25-dihydroxyvitamin D3 (Majore et al., 2011). It should be also noted that the herein presented study employed a comparable ucMSC isolation procedure but used antibiotics and human serum for isolation and differentiation.

Furthermore, spheroids derived from adMSCs and bmMSCs revealed incremental increase of calcium deposition, indicating successful osteogenic differentiation. However, this was not reflected by the ALP activity, which appeared to decrease from day 3 onwards. One possible explanation for this phenomenon might be the increasing compactness of the spheroids during differentiation, which potentially exacerbated the formation of calcium phosphate crystals, that might have trapped the secreted molecules within the spheroid. A similar effect was previously described by (Graham et al., 2020) for adipokine release in adipose spheroids.

Comparing the cell populations included in this study, bmMSCs showed a slightly higher chondrogenic differentiation potential compared to other MSC sources, which was also reported in previous studies (Kohli et al., 2015; Reinisch et al., 2015; Mohamed-Ahmed et al., 2021). Furthermore, adMSCs exhibited superior adipogenic and osteogenic lineage commitment compared to bmMSCs and ucMSCs. Although this seems to be contradictory to the general acceptance of bmMSCs to perform best during adipogenesis and osteogenesis, there is a number of reports outlining contradicting tendencies (Baksh et al., 2007; Gao et al., 2014). In this context, it cannot be excluded that the combined application of advanced culturing conditions in the presented system affects MSC differentiation capacity. Although there is cumulative evidence to suggest tissue specific lineage tendencies, further studies are required to explore the exact cause for the mentioned phenomena and systematically investigate the interplay and impact of hypoxia, and serum- and antibiotics-free media applied on MSC spheroids and their trilineage differentiation depending on the tissue source.

Despite promising results, certain aspects of the herein presented advanced trilineage differentiation system can be improved. In this regard, although employment of single donors is sufficient for the initial establishment of such experimental platforms, in the future transcriptomic data analysis and the use of multiple donors per tissue source as a cell pool or separately examined would provide better insights into the biological variability and overall reliability of the readouts. The variability can be caused by donor-specific variations including sex, age and environmental factors should be considered in the interpretation of future results. It is also crucial to note that a comparison of our work with the cited references reveals certain limitations. These studies employ the aforementioned culture conditions only to a limited extent or not at all. Nevertheless, a comparison is still worthwhile, provided that the discrepancy is considered.

This study has established a scaffold-free trilineage 3D differentiation platform under advanced culture conditions for MSCs derived from bone marrow, adipose and umbilical cord. We characterized samples from this platform model by evaluating spheroid diameters as well as assessing differentiation potential by examining differentiation-specific ECM component accumulation and expression of selected released differentiation markers. Our model showed consistent spheroid growth throughout the differentiation process for all MSC sources. Furthermore, this model promoted successful trilineage differentiation, as indicated by the deposition of lineage-specific ECM and the secretion of differentiation markers, except for osteogenesis of ucMSCs. In the future, this platform can be used to generate *in vitro* microtissues, building blocks for tissue engineering or as a disease model. A potential application in this regard might be studying the histological perturbance of bone sarcomas, including osteosarcoma, chondrosarcoma and Ewing sarcoma during the course of differentiation. In comparison to existing bone sarcoma 3D models (Molina et al., 2020; Monteiro et al., 2021; Freeman et al., 2022), the herein presented platform might provide superior reliability due to the applied physiological conditions. Those factors comprising scaffold-free three-dimensional cultivation, hypoxia, serum-free and antibiotics-free media aim to recapitulate the human *in vivo* microenvironment and thus to increase the relevance of data acquired from this system and facilitate clinical translation in the future.

## 5 Patient consent statement

All three patients gave written consent.

## Data Availability

The raw data supporting the conclusions of this article will be made available by the authors, without undue reservation.

## Abbreviations

2D, Two-dimensional; 3D, Three-dimensional; adMSC, Human adipose tissue-derived MSCs; ALP, Alkaline phosphatase; ANOVA, two-way analysis of variance; bmMSCs, Human bone marrow-derived MSCs; DMMB, 1,9-dimethylmethylene blue; ECM, Extracellular matrix; FBS, Fetal bovine serum; hPL, Human platelet lysate; MSCs, Human mesenchymal stem/stromal cells; PBS, Phosphate-buffered saline; sGAG, Sulfated glycosaminoglycans; ucMSCs, Human umbilical cord-derived MSCs.
