# Supplementary figures and images for: Spheroid trilineage differentiation model of primary mesenchymal stem/stromal cells under hypoxia and serum-free culture conditions
Source: Front Bioeng Biotechnol. 2024 Jul 31;12:1444363. doi: 10.3389/fbioe.2024.1444363 (PMC11321963; doi:10.3389/fbioe.2024.1444363)

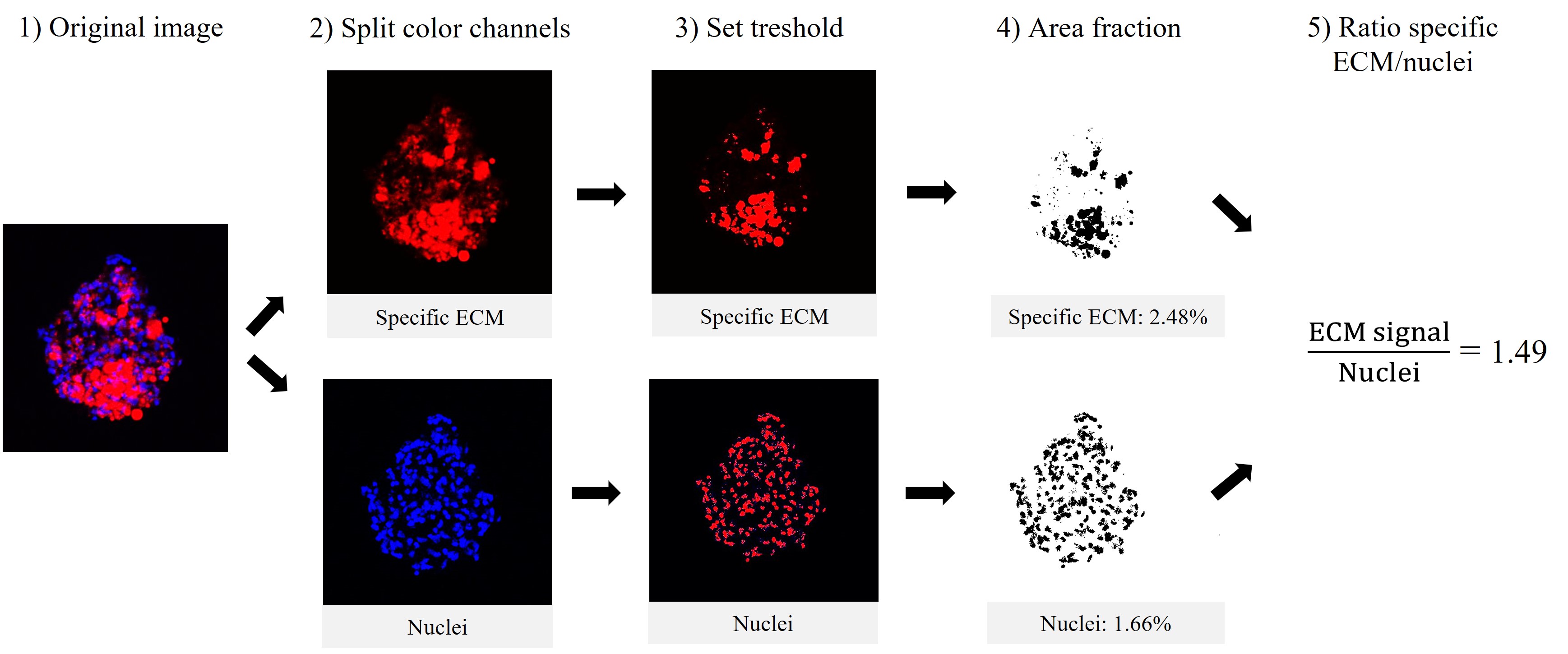

Supplement: Supplementary file 1 [file Image3.JPEG]

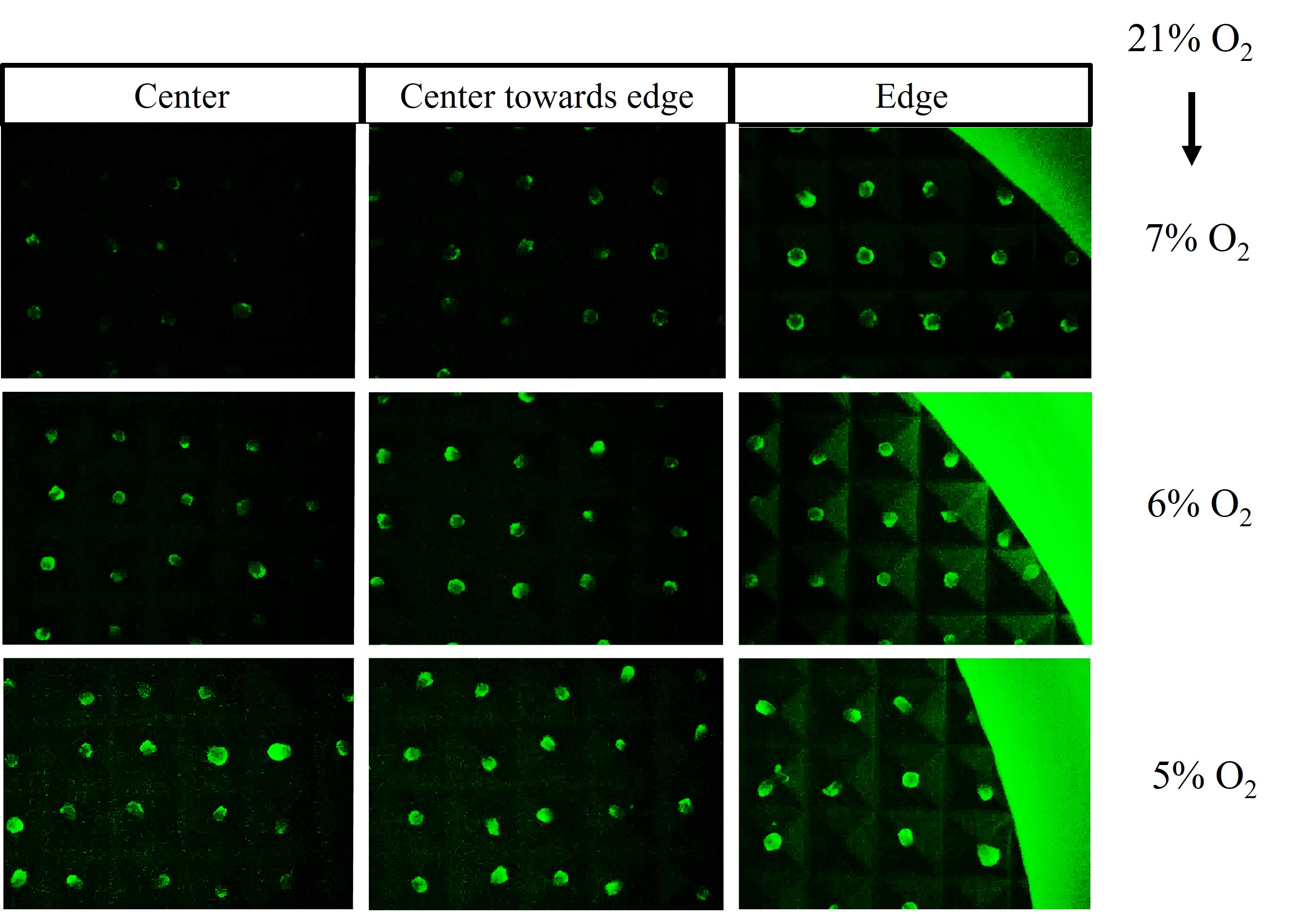

Supplement: Supplementary file 2 [file Image1.JPEG]

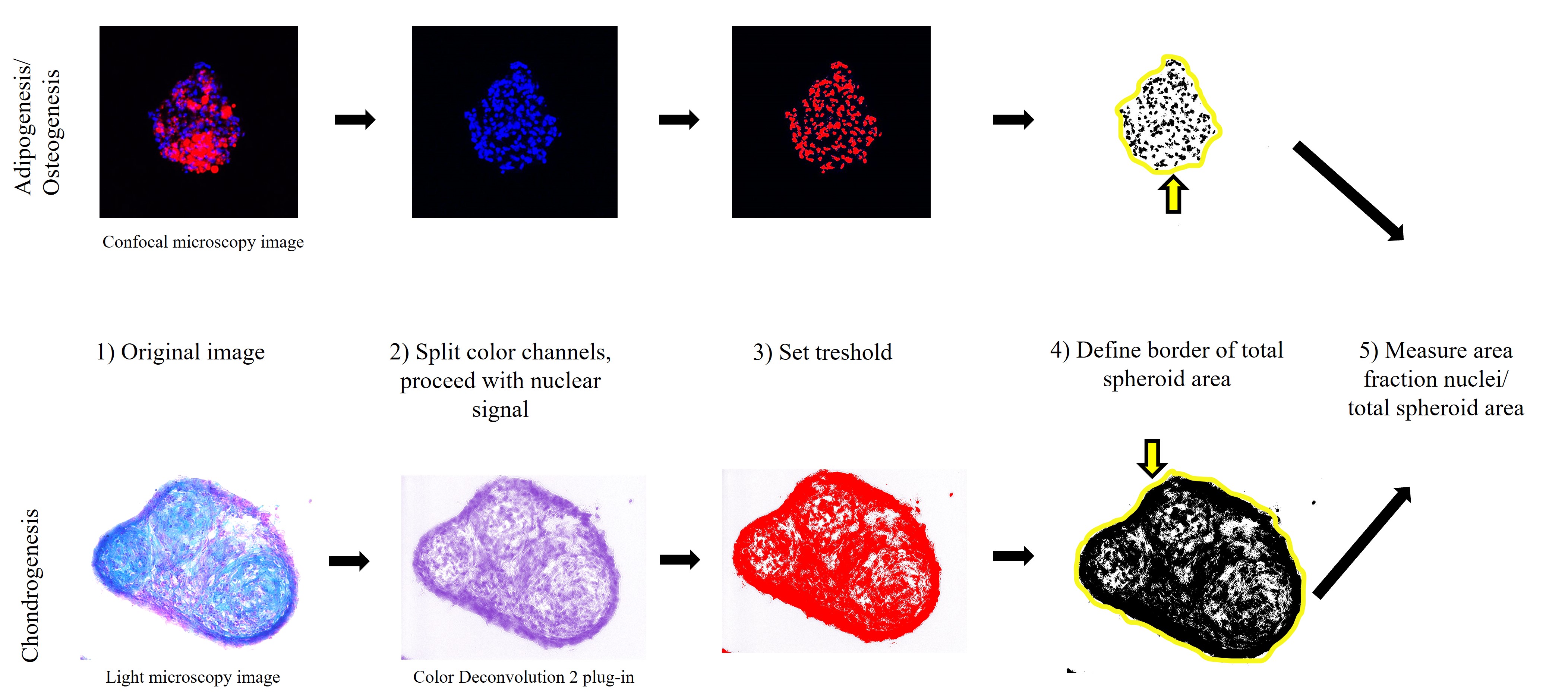

Supplement: Supplementary file 3 [file Image2.JPEG]
