# Supplementary material for: Spheroid trilineage differentiation model of primary mesenchymal stem/stromal cells under hypoxia and serum-free culture conditions
Source: Front Bioeng Biotechnol. 2024 Jul 31;12:1444363. doi: 10.3389/fbioe.2024.1444363 (PMC11321963; doi:10.3389/fbioe.2024.1444363)
Supplement: Supplementary file 4 [file DataSheet1.pdf]

Supplementary Material

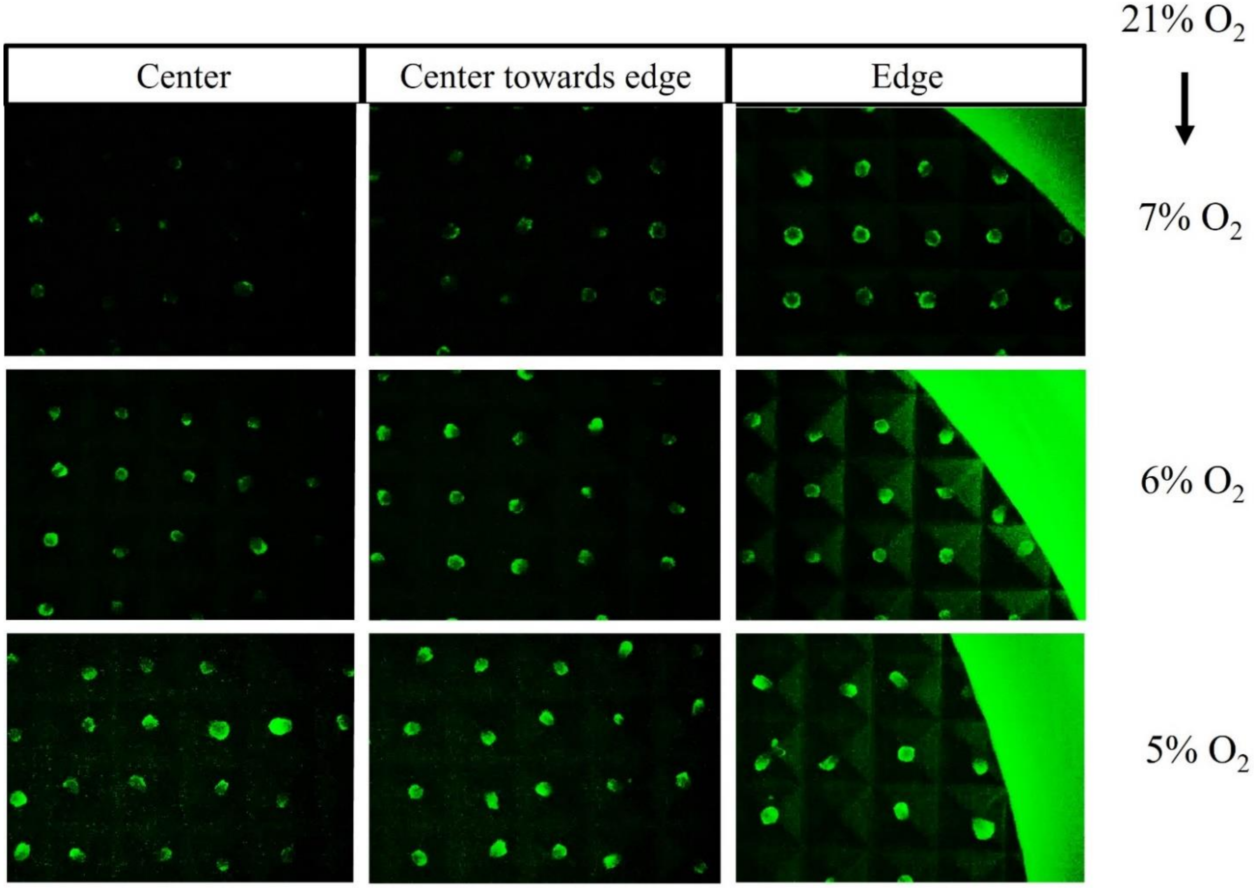

Supplementary Figure 1. Preliminary hypoxia onset measurements of spheroids in the employed Sphericalplate 5D® low attachment micropatterned 24-well plates under different hypoxic conditions. HRE-MSC were used, which are based on the stabilization of hypoxia inducible factor 1α (HIF-1α) upon hypoxia, leading to the expression of the green fluorescent protein UnaG. The reporter cells were utilized to visualize hypoxia onset in the spheroids. Starting at 21% O_2, the oxygen level was gradually reduced. At 5% O_2, a strong and evenly distributed fluorescence signal of the reporter cells was observed for the first time, indicating the onset of hypoxia. Based on these observations, the hypoxic oxygen level for the experiment was determined to be 5% O_2.

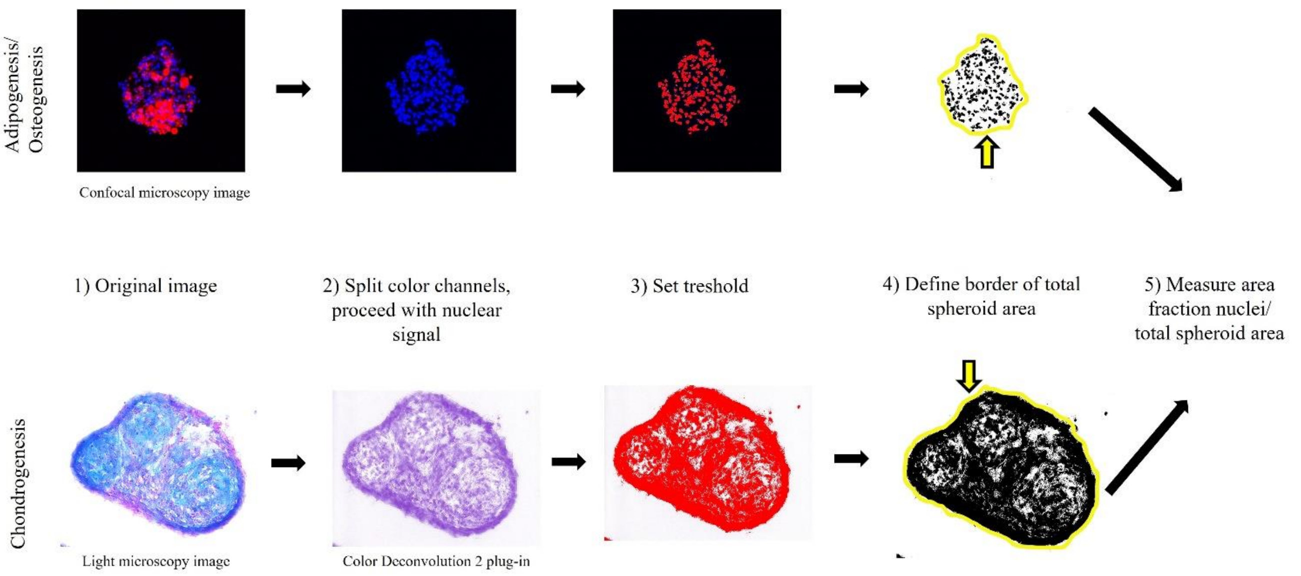

Supplementary Figure 2. Workflow for semiquantitative image analysis of area fraction nuclei/ total spheroid in ImageJ. For adipogenic and osteogenic differentiation, original confocal images were imported into ImageJ, while for chondrogenesis light microscope images were processed. All image stacks were previously acquired with a 20x magnification objective at a resolution of 1024 × 1024 pixels, scan speed 5µs, z-step size of 2 µm across 60 µm. As the second step, colors were split and the image showing the nuclei signal channel was further processed. The nuclei signal for adipogenic and osteogenic differentiation is DAPI, for chondrogenesis Nuclear Fast Red. For light microscopy pictures, the *Color Deconvolution 2* plug-in was employed. In the third step, the threshold was determined for the nuclei signal. For DAPI 95/255 and for Nuclear Fast Red 0/200. The border of the total spheroid area was manually determined. Based on this, the area fraction % of the nuclei signal was measured in µm. For the confocal images, the area fraction of the nuclei signal to the total spheroid was measured separately for each z-stack image and the obtained values of all z-stacks were averaged for the complete image. Three complete images per sample were processed and the values obtained were averaged. Subsequently, a maximum projection of all z-stacks was generated for clear visualization, as shown in Figure 3A, 3B, and 3C.

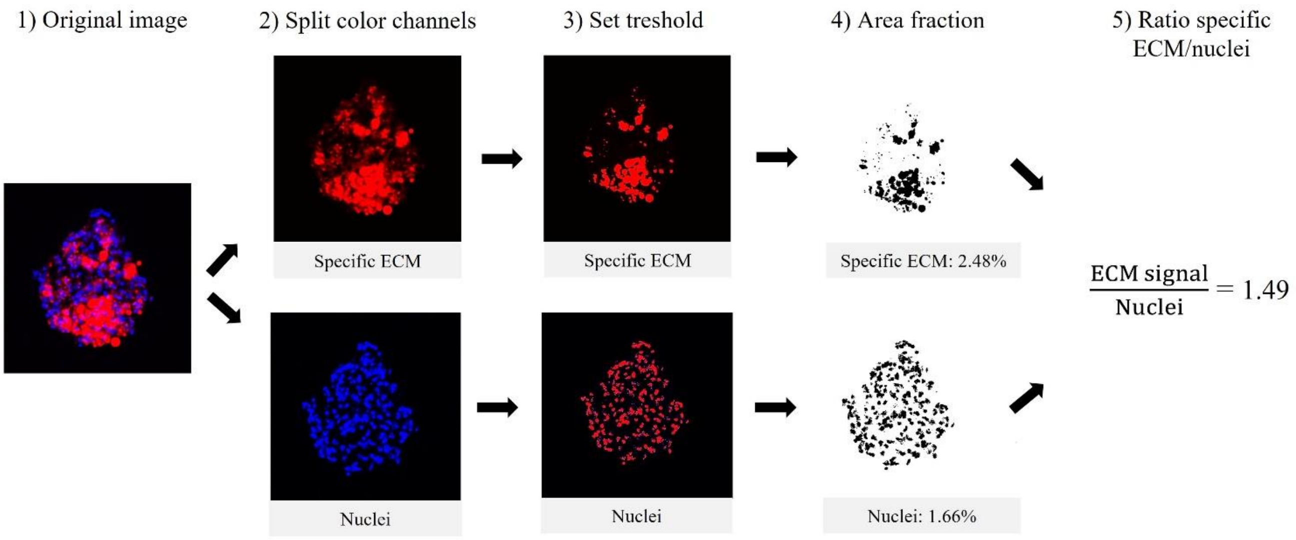

Supplementary Figure 3. Workflow for semiquantitative image analysis of specific ECM/ nuclei signal ratios in ImageJ. Original microscope pictures were imported in ImageJ as described in Supplementary Figure 2. All image stacks were previously acquired with a 20x magnification objective at a resolution of 1024 × 1024 pixels, scan speed 5µs, z-step size of 2 µm across 60 µm. In the next step, color channels were split using the methods described above. In the third step, the threshold for each channel was determined. The threshold for the Nile Red signal (adipogenic differentiation) was set at 35/255, for Calcein (osteogenic differentiation) at 50/255, for Alcian Blue (chondrogenic differentiation) at 0/200. The threshold for the nuclei signals DAPI and Nuclear Fast Red was set as described in Supplementary Figure 2. For light microscopy pictures, the *Color Deconvolution 2* plug-in was employed. The signals were measured and the area ratio (in µm) was then calculated by dividing the area % of the specific ECM signal by the area % of the nuclei signal, which served as background. For the confocal images, the ratio of specific ECM signal to nuclei was measured separately for each z-stack image for both channels in parallel and the obtained values of all z-stacks were averaged for the complete image. Three complete images per sample were processed and the values obtained were averaged. Subsequently, a maximum projection of all z-stacks of both channels was generated for clear visualization, as shown in Figure 3A, 3B, and 3C.
